# Aerobic fitness is associated with greater white matter integrity in children

**DOI:** 10.3389/fnhum.2014.00584

**Published:** 2014-08-19

**Authors:** Laura Chaddock-Heyman, Kirk I. Erickson, Joseph L. Holtrop, Michelle W. Voss, Matthew B. Pontifex, Lauren B. Raine, Charles H. Hillman, Arthur F. Kramer

**Affiliations:** ^1^Department of Psychology, University of Illinois at Urbana-ChampaignUrbana, IL, USA; ^2^Beckman Institute for Advanced Science and Technology, University of Illinois at Urbana-ChampaignUrbana, IL, USA; ^3^Department of Psychology, University of PittsburghPittsburgh, PA, USA; ^4^Department of Bioengineering, University of Illinois at Urbana-ChampaignUrbana, IL, USA; ^5^Department of Psychology, University of IowaIowa City, IA, USA; ^6^Department of Kinesiology, Michigan State UniversityEast Lansing, MI, USA; ^7^Department of Kinesiology and Community Health, University of Illinois at Urbana-ChampaignUrbana, IL, USA

**Keywords:** cardiorespiratory fitness, development, diffusion tensor imaging, fiber tracts, microstructure

## Abstract

Aerobic fitness has been found to play a positive role in brain and cognitive health of children. Yet, many of the neural biomarkers related to aerobic fitness remain unknown. Here, using diffusion tensor imaging, we demonstrated that higher aerobic fitness was related to greater estimates of white matter microstructure in children. Higher fit 9- and 10-year-old children showed greater fractional anisotropy (FA) in sections of the corpus callosum, corona radiata, and superior longitudinal fasciculus, compared to lower fit children. The FA effects were primarily characterized by aerobic fitness differences in radial diffusivity, thereby raising the possibility that estimates of myelination may vary as a function of individual differences in fitness during childhood. White matter structure may be another potential neural mechanism of aerobic fitness that assists in efficient communication between gray matter regions as well as the integration of regions into networks.

## INTRODUCTION

Strategies to optimize cognitive and brain development, including physical activity, computerized training games, martial arts, yoga, mindfulness, and school curricula, are in the spotlight (Diamond and Lee, 2011). For example, aerobic fitness plays an important role in the brain health of children (see Chaddock et al., 2011 for a review), especially in terms of brain structure and brain function, including gray matter volumes [e.g., hippocampus (Chaddock et al., 2010a), basal ganglia (Chaddock et al., 2010b)] and functional brain networks (e.g., frontal and parietal cortex; Voss et al., 2011; Chaddock et al., 2012a). These fitness-related differences in brain health are often coupled with performance differences, such that higher fit children have been shown to outperform their lower fit peers on tasks of cognitive control (Chaddock et al., 2010b, 2012a; Voss et al., 2011) and memory (Chaddock et al., 2010a) as well as scholastic achievement tests in the classroom (Coe et al., 2006; Castelli et al., 2007; Trudeau and Shephard, 2008; Chomitz et al., 2009).

Still unknown is the relationship between aerobic fitness and the microstructure of white matter tracts during childhood. The structure of white matter is important for efficient transmission of information between gray matter as well as for the integration of brain areas into structural networks to support cognitive function. During childhood, many white matter tracts increase in estimates of microstructure, in parallel with improvements in cognition (Barnea-Goraly et al., 2005; Muetzel et al., 2008; see Schmithorst and Yuan, 2010, for a review), but there has been little research about individual differences in the structure of these pathways, the plasticity of white matter fibers, or their susceptibility to intervention during development. Given that the size and function of several different gray matter regions differ between higher fit and lower fit children (Hillman et al., 2009; Chaddock et al., 2010a,b; Davis et al., 2011; Pontifex et al., 2011; Voss et al., 2011; Chaddock et al., 2012a), it is possible that white matter microstructure that connects gray matter areas also differs as a function of aerobic fitness. In support of such a hypothesis, higher aerobic fitness levels and participation in physical activity are positively associated with white matter structure in older adults (Colcombe et al., 2006; Marks et al., 2007; Johnson et al., 2012; Voss et al., 2012).

Here, for the first time, we used diffusion tensor imaging (DTI) to investigate the microstructural properties of white matter in higher fit and lower fit 9- and 10-year-old children. We analyzed fractional anisotropy (FA), a general index of white matter microstructure, hypothesized to be higher in tightly bundled, structurally compact fibers with high integrity (Basser, 1995; Beaulieu, 2002; Sen and Basser, 2005; Rykhlevskaia et al., 2008). We also explored specific patterns of diffusivity [radial diffusivity (RD) and axial diffusivity (AD)], hypothesized to reflect potential biological properties of white matter microstructure (Basser, 1995; Pierpaoli and Basser, 1996; Pierpaoli et al., 1996; Song et al., 2002). For example, a reduction in RD is observed in the presence of remyelination, causing RD to often be used as a marker of myelination (Song et al., 2002, 2003, 2005; Nair et al., 2005; Budde et al., 2007; Rykhlevskaia et al., 2008). Additionally, AD is said to be sensitive to changes in axonal fibers including axonal diameter, loss or damage (Song et al., 2003; Budde et al., 2007). By exploring the associations among aerobic fitness and measures of diffusivity (FA, RD, AD), we can investigate microstructural white matter properties influenced by aerobic fitness during child development.

We predicted that higher fit children would show greater estimates of white matter microstructure than lower fit children. Because the structure and function of brain circuits important for cognitive control have been found to be sensitive to aerobic fitness (Bunge and Crone, 2009; see Chaddock et al., 2012b for a review), we focused on white matter tracts that relate to these cognitive skills (Liston et al., 2006; Madsen et al., 2010; Tamnes et al., 2012; Chaddock-Heyman et al., 2013), and that connect frontal, striatal and parietal brain areas. In particular, in this first exploration of the association between aerobic fitness and white matter microstructure in children, we examined a *priori* white matter tracts (included in Chaddock-Heyman et al., 2013). These tracts included the corpus callosum, which connects the left and right cerebral hemispheres (Olesen et al., 2003; Nagy et al., 2004; Tamnes et al., 2012), the corona radiata which carries ascending and descending information throughout the brain (Olesen et al., 2003; Bendlin et al., 2010; Niogi et al., 2010; Chaddock-Heyman et al., 2013), and the superior longitudinal fasciculus, a tract that provides bidirectional information transfer between the frontal and parietal cortex (Nagy et al., 2004; Chaddock-Heyman et al., 2013). Additionally, we examined the posterior thalamic radiation, nerve fibers connecting the thalamus and caudate nucleus with the cerebral cortex (Olson et al., 2009; Chaddock-Heyman et al., 2013), and the cerebral peduncle, part of the brainstem, which includes nerve tracts carrying motor information to and from the brain to the rest of the body (Olson et al., 2009; Chaddock-Heyman et al., 2013). The present study contributes to our understanding of plasticity of the microstructure of white matter that allows efficient communication between gray matter areas in the brain and integrates the brain into networks important for cognition and academic achievement.

## MATERIALS AND METHODS

### PARTICIPANTS

Preadolescent 9- and 10-year-old children were recruited from East-Central Illinois. Children were screened for several factors that influence physical activity participation or cognitive function. To begin, the Kaufman Brief Intelligence Test (K-BIT; Kaufman and Kaufman, 1990) was administered to each child to obtain a composite intelligence quotient (IQ) score including both crystallized and fluid intelligence measures. Participants were excluded if their scores were more than 1 standard deviation below the mean (85%). Next, a guardian of the child completed the attention-deficit hyperactivity disorder (ADHD) Rating Scale IV (DuPaul et al., 1998) to screen for the presence of attentional disorders. Participants were excluded if they scored above the 85th percentile. Pubertal timing was also assessed using a modified Tanner Staging System (self-report, with schematics; Taylor et al., 2001) with all participants at or below a score of 2 on a 5-point scale of developmental stages. In addition, socioeconomic status (SES) was determined by creating a trichotomous index based on three variables: participation in a free or reduced-price meal program at school, the highest level of education obtained by the child’s mother and father, and the number of parents who worked full-time (Birnbaum et al., 2002).

Furthermore, eligible participants were required to (1) qualify as higher fit or lower fit (see Aerobic Fitness Assessment), (2) demonstrate right handedness (as measured by the Edinburgh Handedness Questionnaire; Oldfield, 1971), (3) report no adverse health conditions, physical incapacities, or neurological disorders, (4) report no use of medications that influenced central nervous system function, (5) successfully complete a mock MRI session to screen for claustrophobia in an MRI machine, and (6) sign an informed assent approved by the University of Illinois at Urbana-Champaign. A legal guardian also provided written informed consent in accordance with the Institutional Review Board of the University of Illinois at Urbana-Champaign.

Fifty-two participants were initially eligible for the present study (after exclusions due to K-BIT scores, ADHD, pubertal timing, VO_2_max criteria, etc). Of this sample, 32 children completed the white matter imaging component of the MRI session. Eight children were excluded from analysis due to visible motion on the reconstructed data and/or lack of whole-brain coverage during acquisition. Twenty-four children were included in the analysis, including 12 higher fit participants (seven boys, five girls) and 12 lower fit participants (eight boys, four girls). All participants were compensated $10/h for the demographic and fitness testing and $20 for participation in the MRI session.

### AEROBIC FITNESS ASSESSMENT

The aerobic fitness level of each child was determined by measuring maximal oxygen uptake (VO_2_max) using a computerized indirect calorimetry system (ParvoMedics True Max 2400) during a modified Balke protocol (American College of Sports Medicine, 2006). Specifically, participants ran on a motor-driven treadmill at a constant speed with increases in grade increments of 2.5% every 2 min until volitional exhaustion. Averages for oxygen uptake (VO_2_) and respiratory exchange ratio (RER; the ratio between carbon dioxide and oxygen) were assessed every 30 s. In addition, heart rate was measured throughout the fitness test [using a Polar heart rate monitor (Polar WearLink^®;^+ 31, Polar Electro, Finland)], and ratings of perceived exertion were assessed every 2 min using the children’s OMNI scale (Utter et al., 2002).

VO_2_max was defined when oxygen consumption remained at a steady state despite an increase in workload. Relative peak oxygen consumption was based upon maximal effort as evidenced by (1) a plateau in oxygen consumption corresponding to an increase of <2 mL/kg/min despite an increase in workload, (2) a peak heart rate >185 beats per minute (American College of Sports Medicine, 2006) accompanied by a heart rate plateau (i.e., an increase in work rate without a concomitant increase in heart rate, Freedson and Goodman, 1993), (3) RER >1.0 (Bar-Or, 1983), and/or (4) ratings on the children’s OMNI scale of perceived exertion >8 (Utter et al., 2002). Relative peak oxygen consumption was expressed in mL/kg/min.

Aerobic fitness group assignments (i.e., higher fit and lower fit) were based on whether a child’s VO_2_max value fell above the 70th percentile or below the 30th percentile according to normative data provided by Shvartz and Reibold (1990). Children who did not qualify as higher fit or lower fit were excluded from further participation.

### MAGNETIC RESONANCE IMAGING ACQUISITION

Diffusion-weighted images were acquired on a Siemens Allegra 3T head-only scanner with repetition time (TR) = 4.8 s, echo time (TE) = 100.4 ms, and 3.44 mm^2^ in-plane resolution with 4 mm slice thickness. To obtain whole-head coverage, 32 slices were collected parallel to the anterior-posterior commissure plane with no interslice gap. Four T2-weighted images (*b*-value = 0 s/mm^2^) and one 30-direction diffusion-weighted echo planar imaging scan (*b*-value = 1000 s/mm^2^) were collected.

Diffusion in the brain can be modeled by a diffusion tensor/diffusion ellipsoid. FA is calculated from the three eigenvalues (λ1, λ2, λ3) of the diffusion tensor and represents the anisotropy (directional dependence) of diffusion (Basser, 1995; Beaulieu, 2002; Sen and Basser, 2005), independently of the rate of diffusion. FA ranges from 0 to 1, with higher values reflecting increased directionality of diffusion (i.e., water traveling more parallel to a tract compared to perpendicularly). In a region with free diffusion, the FA value is 0 and the diffusion is isotropic. If the diffusion is more in one direction, i.e., anisotropic diffusion, the FA value approaches 1. RD is the average of the second and third eigenvalues (λ2, λ3), reflective of diffusivity perpendicular to the major axis of the tensor (Basser, 1995; Pierpaoli and Basser, 1996; Pierpaoli et al., 1996; Song et al., 2002). AD is the diffusion along the principal diffusion eigenvalue (λ1) of the ellipsoid.

### DIFFUSION DATA ANALYSIS

Image analyses and tensor calculations were performed using FSL 5.0.1 (FMRIB Software Library). First, each participant’s data were passed through an automated pipeline consisting of (1) motion and eddy current correction, (2) removal of non-brain tissue using the Brain Extraction Tool (Smith, 2002), and (3) local fitting of the diffusion tensor model at each voxel using FMRIB’s Diffusion Toolbox v2.0 (FDT)^1^. The products of the multi-step pipeline included FA and AD images; RD maps were calculated as the mean of the second and third eigenvalues (Song et al., 2002).

Next, diffusion data were processed using TBSS v1.2 (Tract-Based Spatial Statistics; Smith et al., 2006). Each participant’s FA data were aligned into the 1 mm × 1 mm × 1 mm standard Montreal Neurological Institute (MNI152) space via the FMRIB58_FA template using the FMRIB’s Non-linear Registration Tool (Andersson et al., 2007a,b), and a mean diffusion image was created. The mean FA image was then thinned to create an average skeleton representing the centers of the tracts shared by all participants, and the skeleton was thresholded at FA > 0.20. Each participant’s aligned FA data were projected onto the skeleton, taking on the FA value from the local center of the nearest relevant tract. RD and AD skeletons for each participant were formed in a similar manner by projecting the analogous data onto the mean skeleton.

### REGION-OF-INTEREST ANALYSIS

**FIGURE 1:**
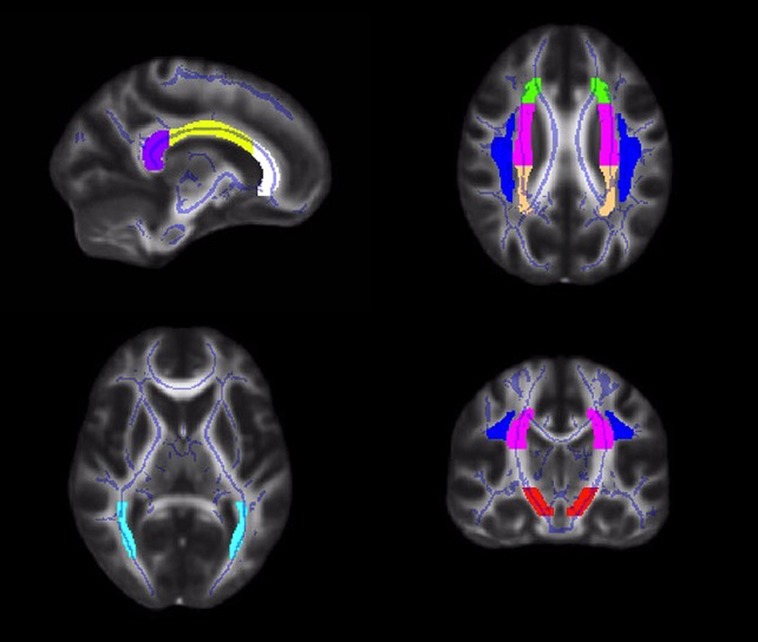
**Illustrations of the white matter tract ROIs in the corpus callosum (white = genu; yellow = body; purple = splenium), corona radiata (anterior = green; superior = pink; posterior = peach), superior longitudinal fasciculus (blue), posterior thalamic radiation (light blue), and cerebral peduncle (red)**.

Diffusion values (FA, RD, AD) were calculated for each participant within *a priori* ROIs, created from the JHU ICBM-DTI-81 white matter labels atlas^2^ (Mori et al., 2005; Wakana et al., 2007; Hua et al., 2008). Tract ROIs were created in the genu, body and splenium of the corpus callosum, and the left and right corona radiata, superior longitudinal fasciculus, posterior thalamic radiation, and cerebral peduncle (**Figure 1**). An FSL command, fslmaths, was used to create each ROI (e.g., fslmaths JHUAtlas –uthr 16 –thr 16 RCerebralPeduncle).

### STATISTICAL ANALYSES

Our primary hypothesis was that higher fit children would show greater white matter microstructure in all tracts of interest, as indexed by FA, compared to lower fit children. First, because there were significant and robust correlations between hemispheres for each tract (all *p* > 0.8, *p* < 0.0001), we averaged FA values across left and right hemispheres. We then performed a multivariate analysis of variance (MANOVA) to examine associations between aerobic fitness group (lower fit, higher fit) and FA in all tract ROIs (genu, body, and splenium of the corpus callosum, average corona radiata, average superior longitudinal fasciculus, average posterior thalamic radiation, average cerebral peduncle). Given a significant multivariate effect, univariate ANOVAs were conducted to examine specific differences between higher fit and lower fit children in FA for each tract of interest. For those tracts showing significant differences in FA (*p* < 0.05) between groups, we conducted secondary analyses on RD and AD to better understand the underlying biological properties of overall FA differences in white matter microstructure (Burzynska et al., 2010; Johnson et al., 2012).

## RESULTS

### PARTICIPANT DEMOGRAPHICS

Demographic and fitness data are provided in **Table 1**. No differences in age, gender, IQ, ADHD, pubertal timing, or SES existed between aerobic fitness groups (*t*’s < 1.4, *p*’s > 0.19). We confirmed that higher fit children had higher relative VO_2_max scores than lower fit children [*t* (22) = 8.0, *p* < 0.001], as expected with our recruitment of extreme aerobic fitness groups.

**Table 1:** Participant mean demographic and fitness data (SD) by aerobic fitness group.

Variable	Lower fit	Higher fit
*N*	12 (8 boys)	12 (7 boys)
Age (years)	10.1 (0.7)	9.9 (0.6)
VO_2_max (mL/kg/min)	35.7 (6.0)^*^	52.1 (3.9)^*^
VO_2_max percentile (%)	14.8 (21.8)^*^	82.7 (3.6)^*^
Height (inches)	141.1 (9.1)	141.5 (5.9)
Weight (pounds)	42.5 (10.1)^*^	33.5 (6.0)^*^
K-BIT^a^ composite score (IQ)	110.6 (15.0)	114.8 (6.8)
K-BIT^a^ crystallized score (vocabulary)	106.4 (12.5)	108.1 (5.5)
K-BIT^a^ fluid score (matrices)	112.2 (17.0)	118.7 (8.8)
ADHD^b^	7.6 (5.5)	6.8 (4.2)
Tanner^c^	1.8 (0.4)	1.5 (0.5)
SES^d^ (median)	3.0 (0.0)	2.7 (0.7)

a Kaufman Brief Intelligence Test (Kaufman and Kaufman, 1990). ^b^Scores on the *ADHD Rating Scale V* (DuPaul et al., 1998). ^c^Pubertal timing assessed using a modified Tanner Staging System (Taylor et al., 2001). ^d^Socioeconomic Status was determined by the creation of a trichotomous index based on three variables: child participation in a free or reduced-price lunch program at school, the highest level of education obtained by the child’s mother and father, and the number of parents who worked full-time (Birnbaum et al., 2002). ^*^Significantly different at *p* < 0.001.

### AEROBIC FITNESS AND WHITE MATTER MICROSTRUCTURE

The overall multivariate test indicated a significant effect of aerobic fitness on white matter microstructure [*F*(9,14) = 4.40, *p* = 0.007]. Next, univariate ANOVAs were performed to identify the specific dependent variables that contributed to the overall effect.

Higher fit children showed greater FA in the body of the corpus callosum [*F*(1,22) = 5.27, *p* = 0.032], bilateral superior corona radiata [*F*(1,22) = 6.18, *p* = 0.021], and bilateral superior longitudinal fasciculus [*F*(1,22) = 5.71, *p* = 0.026; **Table 2**]. No aerobic fitness differences were found in FA of the genu of the corpus callosum [*F*(1,22) = 1.51, *p* = 0.23], splenium of the corpus callosum [*F*(1,22) = 2.09, *p* = 0.16], anterior corona radiata [*F*(1,22) = 0.34, *p* = 0.57], posterior corona radiata [*F*(1,22) = 2.37, *p* = 0.08], posterior thalamic radiation [*F*(1,22) = 1.11, *p* = 0.30] or cerebral peduncle [*F* (1,22) = 0.23, *p* = 0.64; **Table 2**].

**Table 2:** FA, RD, and AD (mean, standard deviation) in white matter tracts of interest in lower fit and higher fit children.

	Lower fit (*M*, SD)	Higher fit (*M*, SD)
FA corpus callosum genu	0.5277 (0.04)	0.5600 (0.08)
FA corpus callosum body	0.4125 (0.03)*	0.4510 (0.05)*
FA corpus callosum splenium	0.5644 (0.02)	0.5789 (0.02)
FA anterior corona radiata	0.3627 (0.03)	0.3763 (0.07)
FA superior corona radiata	0.3441 (0.02)*	0.3749 (0.04)*
FA posterior corona radiata	0.3149 (0.02)	0.3292 (0.02)
FA superior longitudinal fasciculus	0.3135 (0.01)*	0.3301 (0.02)*
FA posterior thalamic radiation	0.3971 (0.02)	0.4076 (0.03)
FA cerebral peduncle	0.4792 (0.07)	0.4684 (0.04)
RD corpus callosum body	1.000 (0.70)	0.900 (0.16)
RD superior corona radiata	0.854 (0.05)*	0.781 (0.10)*
RD superior longitudinal fasciculus	0.928 (0.04)*	0.871 (0.07)*
AD Corpus callosum body	1.914 (0.12)	1.87967 (0.18)
AD superior corona radiata	1.486 (0.08)	1.425 (0.11)
AD superior longitudinal fasciculus	1.459 (0.06)	1.413 (0.09)

RD and AD values are reported in μm^2^/ms. *Significant aerobic fitness group difference, *p* < 0.05.

Secondary analyses on these significant regions showed that group differences in FA in the body of the corpus callosum were not driven by significant differences in RD or AD (*F*’s < 2.3, *p’*s > 0.1). Fitness differences in FA in the superior corona radiata were driven by significant differences in RD [*F*(1,22) = 4.97, *p* = 0.036]. Additionally, aerobic fitness differences in the superior longitudinal fasciculus were characterized by group differences in RD [*F*(1,22) = 6.61, *p* = 0.017]. There were no aerobic fitness group differences in AD in any white matter tracts. **Table 2** provides means and standard deviations for the DTI measures of white matter microstructure for higher fit and lower fit children.

## DISCUSSION

The present study is the first to demonstrate that aerobic fitness is positively related to the microstructure of white matter fiber tracts in the brain during childhood. Higher fit 9- and 10-year-old children (>70th percentile VO_2_max) showed greater FA in sections of the corpus callosum, corona radiata, and superior longitudinal fasciculus, compared to lower fit children (<30th percentile VO_2_max). An exploration of the components of the diffusion tensor showed that the relationships between aerobic fitness and FA were primarily characterized by fitness group differences in RD. Because axonal caliber and thickness of the myelin sheath determine conduction velocity (Paus, 2010), our results raise the possibility that children with higher fitness levels may have faster neural conduction between brain regions important for cognitive control. White matter microstructure may be another potential neural mechanism of aerobic fitness that assists in efficient communication between gray matter and the integration of regions into networks to support cognitive function and academic achievement.

The analysis of different patterns of diffusivity allows us to speculate about the biological properties of white matter structure that may differ as a function of aerobic fitness in children. First, our data suggest that higher fit children have greater FA, or more tightly bundled and structurally compact fibers, in the corpus callosum, specifically in the body of the white matter tract. This effect in children supports and extends the association between aerobic fitness and the body of the corpus callosum in an aging population (Johnson et al., 2012). Whereas the fitness-FA association in the corpus callosum in older adults was driven by negative fitness-RD associations (Johnson et al., 2012), we did not observe aerobic fitness group differences in RD or AD in this tract in the child sample. Thus, it is possible that the increased estimate of fiber integrity in this tract was not solely a function of greater myelination (RD) or axonal fiber diameter (AD). Other tissue differences may have played a role, such as increased neurotrophic factors or increased vascularization, two cellular effects of exercise which are observed in rodents that could influence white matter (Ding et al., 2006). Regardless of the biological mechanism, because the corpus callosum connects the left and right cerebral hemispheres and facilitates interhemispheric communication and the exchange of cognitive, motor and sensory information between the hemispheres (Gazzaniga, 1995), structurally compact fibers may help lead to superior cognitive performance in higher fit children, as suggested in previous work (Olesen et al., 2003; Nagy et al., 2004; Tamnes et al., 2012).

We also demonstrate significant effects of aerobic fitness on FA in the superior corona radiata and superior longitudinal fasciculus, driven by associations with RD. We suggest that aerobic fitness during childhood may influence fiber structural integrity and fiber alignment in these tracts perhaps via increased myelination (reductions in RD; Song et al., 2003, 2005; Sun et al., 2006, 2008). To link RD to myelination, Song et al. (2005) used an experimental model of demyelination and remyelination in a mouse brain and demonstrated that RD increased with demyelination and subsequently decreased with the progression of remyelination. The RD changes were found to be specific to changes in myelin integrity, and distinct from axonal injury (Song et al., 2005). Like the corpus callosum, no aerobic fitness differences were found in terms of AD in the superior corona radiata or superior longitudinal fasciculus, which suggests that axonal diameter, loss or damage may not relate to individual differences in fitness (Song et al., 2003; Budde et al., 2007). This is not unexpected given that axonal information is not influenced by myelination (Song et al., 2003; Budde et al., 2007). Because the corona radiata has ascending and descending tracts from the cerebral cortex that integrate information throughout the brain, and cognitively, the tract is known to play a role in processing speed, cognitive control and memory (Olesen et al., 2003; Bendlin et al., 2010; Niogi et al., 2010; Chaddock-Heyman et al., 2013), it is important to investigate its plasticity. Similarly, as the superior longitudinal fasciculus provides bidirectional information transfer between the frontal and parietal cortex (Petrides and Pandya, 1984; Schmahmann and Pandya, 2006), and these connections have been found to correlate with performance on tasks of attentional and interference control (Chaddock-Heyman et al., 2013), and working memory in children (Nagy et al., 2004), our data have promising implications for the influence of aerobic fitness on brain health during childhood.

Whereas we demonstrate that aerobic fitness is associated with white matter microstructure across a number of tracts throughout the brain, the results also demonstrate some specificity. The fitness groups did not differ in the structure of the posterior thalamic radiation or cerebral peduncle, two tracts involved in motor function. Given our recent report of an association between interference control and the microstructure of the posterior thalamic radiation and cerebral peduncle (Chaddock-Heyman et al., 2013), and our reports that higher fit children show less interference RT than lower fit peers (Chaddock et al., 2010b), it is surprising that higher fit children did not have greater estimates of structure in these lower level motor tracts. However, some research suggests that cognitive gains with physical activity and fitness are specific to tasks that require increased cognitive control (Kramer et al., 1999; Colcombe and Kramer, 2003; Colcombe et al., 2004; Chaddock et al., 2011), so it is possible that aerobic fitness would not influence lower level motor tracts, as observed herein.

This study provides a first step in identifying the relationship between aerobic fitness and white matter microstructure during childhood, but the results should be evaluated in the context of their limitations. Studies with larger sample sizes, whole-brain exploratory analyses, a physical activity intervention, and a number of cognitive tasks should be conducted to confirm the complex relationship among physical activity, aerobic fitness, white matter structure, and cognition. Because we approached the study with a *priori* hypotheses, additional investigations should employ whole-brain analyses corrected for multiple comparisons. Also, it is important to remember that DTI does not measure tissue parameters (e.g., fiber integrity, myelination) directly, but measures the displacement of water molecules. Thus, indices of underlying microstructural properties can only be inferred from this displacement. Furthermore, because there are many axons within a voxel and TBSS only selects one axon per voxel, a number of different tissue parameters can result in differences in FA, RD, and AD.

These results arrive at an important time, as children become increasingly unfit and sedentary, and educators reduce or eliminate opportunities for physical activity during the school day in favor of academic topics (Centers for Disease Control and Prevention, 2010). In fact, white matter microstructure in the corona radiata has been found to relate to mathematics performance in the classroom (van Eimeren et al., 2008), which raises the possibility that the removal of fitness training opportunities during the school day may unintentionally have deleterious effects on white matter tracts, potentially reducing scholastic achievement. In fact, our study raises the possibility that greater white matter integrity, perhaps via greater myelination, is one pathway by which higher fit children excel in cognitive and academic performance compared to their less fit peers. Hopefully these findings will reinforce the importance of aerobic fitness during development and lead to additional physical activity opportunities in and out of the school environment.

## Conflict of Interest Statement

The authors declare that the research was conducted in the absence of any commercial or financial relationships that could be construed as a potential conflict of interest.

## Footnotes

1 http://www.fmrib.ox.ac.uk/fsl/fdt

2 http://www.fmrib.ox.ac.uk/fsl/data/atlas-descriptions.html#wm
